# Global Changes in Lepidopteran Phylogenetic Diversity Across Space and Time

**DOI:** 10.1002/ece3.72557

**Published:** 2026-01-12

**Authors:** Jillian Muirberry, Lesley T. Lancaster

**Affiliations:** ^1^ University of Aberdeen, School of Biological Sciences Aberdeen UK

**Keywords:** butterflies and moths, competition, ecological filtering, habitat loss, insect, latitudinal cline, macrogenetics, phylogenetic diversity, range shift

## Abstract

Amidst increasing reports of insect declines, it is ever more important to understand spatial and temporal insect diversity patterns and processes. Phylogenetic diversity (PD) is an important biodiversity metric in that it relates strongly to ecosystem processes, and it can be estimated more accurately from opportunistic occurrence data than other elements of biodiversity. Here, we assess recent changes across global variation in Lepidopteran PD, to discover overall patterns, their repeatability across regions and environmental drivers. We assess global, spatiotemporal variation in PD, as compared to null expectations given sampling effort, determining how such variation relates to region, space, time and environment. Our analysis is based on 374,749 gene sequence accessions from the barcode of life database (BOLD), representing 3158 species assemblages, spanning 62 years. We find that global variation in PD of Lepidopteran species assemblages has significantly increased over time at high latitudes while remaining relatively unchanged near the equator. This pattern exhibits parallelism across global regions, with the strongest increases in PD towards the present observed in high‐latitude communities in North America and Asia, in lowland sites in Europe, and across the African continent. In contrast, PD has declined through time in wetter portions of Australasia and in Africa and South America. Our reported patterns likely reflect changes in Lepidopteran responses to tropical habitat loss and widespread range expansions to higher latitudes. However, changing clines in DNA barcoding strategies could also play a role. Detecting spatiotemporal patterns of change in PD at the global scale is enabled by the increasing use of genetic markers in taxonomy. Our replicated findings provide confidence in biogeographic interpretation, yet increased metadata on sub‐sampling decisions would aid future interpretation of biodiversity trends using ecological genomics synthesis.

## Graphical abstract

Amidst increasing reports of insect declines, it is ever more important to understand spatial and temporal insect diversity patterns and processes. In this study, we repurposed the wealth of sequence data initially used for species identification, to detect global changes in the phylogenetic diversity (PD) of Lepidopteran communities over recent decades. We found evidence of increasing PD in cool temperate regions but declining PD in wet, tropical areas; patterns which likely reflect both tropical habitat loss and widespread range expansions to higher latitudes. Detecting spatiotemporal patterns of change in community PD at the global scale is enabled by the increasing use of genetic markers in taxonomy, and resulting data can inform patterns of ongoing biodiversity shifts.

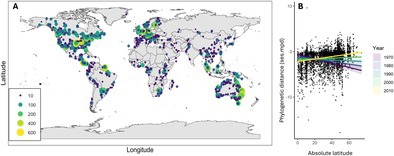

## 1 Introduction

Changes in and especially loss of biodiversity represent a widespread and urgent problem, with changes in land use, exploitation and climate considered to be key drivers. The preservation of biodiversity is a key conservation and management objective, because ecosystem biodiversity often promotes ecological resilience and functional responses to environmental degradation and disturbance (Franklin 1993), as well as having intrinsic value. Biodiversity is a very broad term that covers a spectrum of different levels of ecological diversity including species diversity, genetic diversity, functional diversity and phylogenetic diversity (Naeem et al. 2016). Nonetheless, many ecological studies aiming to quantify biodiversity for management and policy have focussed solely on species richness (Winter et al. 2013; Voskamp et al. 2017). This is potentially problematic and can lead to misleading conclusions about biodiversity dynamics, as it is becoming more apparent that species richness alone is ineffective in describing the full spectrum of biodiversity impacts on ecological systems (Karanth et al. 2019; Gumbs et al. 2020).

Phylogenetic diversity (PD) metrics measure the relatedness of species within a local species assemblage or community, and when coupled with an understanding of the evolutionary conservatism of underlying niche traits, can give insight into the community's evolutionary past, ecosystem interactions and assembly patterns. Where niche traits that impact a population's colonisation success and persistence within a local area are phylogenetically conserved, then ecological conditions may filter for related species that share tolerances to such conditions, while unrelated species are excluded, resulting in patterns of local phylogenetic underdispersion (Webb 2000). In contrast, where key niche traits impacting establishment and persistence are highly evolutionarily labile, then phylogenetic overdispersion may be observed (Mayfield and Levine 2010). Thus, combined with an understanding of how niche traits evolve, estimates of regional variation in local PD and its changes through time can provide an improved understanding of underpinning ecological and evolutionary processes.

Biodiversity is unevenly distributed across the globe, with a general macroecological pattern emerging of increasing species richness with decreasing latitude (Gaston 1996; Willig et al. 2003). However, the spatial distribution of diversity can be highly variable depending on the biogeographic origin of clades and the distribution of habitats (Wiens and Donoghue 2004; Hawkins and DeVries 2009). In addition to understanding the spatial structure of biodiversity, understanding temporal trends has clear value in predicting and mitigating future patterns, especially when evaluating human‐mediated biodiversity declines (Dornelas et al. 2018; Lovell et al. 2023). For instance, many studies have reported a marked decrease in insect species richness and abundance in recent years (Habel et al. 2021; Hallmann et al. 2017; Leather 2018; Seibold et al. 2019; Wagner 2019). This is a great cause for concern, as insects provide a vast range of ecosystem services, including pollination, decomposition, pest control and are a food source for many other species (Sánchez‐Bayo and Wyckhuys 2019). However, the data on insect declines often suffers from a range of biases in part related to the difficulty in comparing species richness across divergent sampling regimes (Cardoso and Leather 2019).

In contrast to species richness estimators, PD can be estimated in ways that are less sensitive to sampling issues, and PD may therefore represent a more practical, as well as more informative, option for cataloguing community diversity on large temporal and spatial scales. Specifically, PD effect sizes measure the extent to which observed levels of phylogenetic clustering within regions, habitats or communities deviate from null expectations, *compared to a random assemblage of the same species richness*. Such PD estimates are, importantly, robust to variation in sampling effort, which varies markedly across latitudes and biomes (Hughes et al. 2021). Therefore, due to its connection to ecosystem functioning, community assembly and other such processes, and its ability to be estimated and compared across different sampling regimes, the use of PD in biodiversity monitoring has many advantages in managing conservation priorities over the use of species richness alone (Vane‐Wright et al. 1991; Crozier 1992).

Many species are currently undergoing rapid range expansions and invasions worldwide (Lenoir et al. 2020), a process which is likely to be evidenced by recent changes in PD where novel, no‐analogue communities are forming (Fitt and Lancaster 2017). In addition, dramatic within‐region global biodiversity loss is ongoing due to recent and rapid habitat loss, pollution and other anthropogenic pressures (IPBES 2019). The use of changing PD as an indicator of ecological regime shift is particularly promising where species' sensitivities to environmental changes are either evolutionarily conserved or overdispersed (Comte et al. 2014). Overall, it is likely that widespread invasions and climate‐mediated range shifts, as well as altered patterns of ecological filtering, competition and extinction risk, should have led to dramatic and recent temporal shifts in the phylogenetic dispersion of impacted communities. Understanding spatial and temporal patterns in the distribution of PD thus may offer support in developing conservation planning strategies (Aguilar‐Tomasini et al. 2021).

Increasingly, studies have begun to investigate large‐scale or global spatial patterns in PD (Fritz and Rahbek 2012; Willig and Presley 2016), with results often showing highest PD, unsurprisingly, in ecotones between biomes, but also in areas characterised by past episodes of range expansion. These results suggest that spatial patterns of PD metrics can provide a very good indicator of recent biogeographic changes. However, we have little direct knowledge of how spatial variation in community PD has changed through time. This has been partly due to the difficulty of performing a large‐scale empirical phylogenetic analysis across global spatiotemporal community sampling events. However, with the advent of global‐scale genetic reference databases such as the Barcode of Life Database (BOLD; Ratnasingham and Hebert 2007), it is now possible to estimate spatiotemporal trends in phylogenetic diversity for numerous animal taxa, based on a commonly used barcode marker gene, mitochondrial cytochrome c oxidase I (mtCOI) (Faith and Baker 2006). Here we leverage global data based on community sampling of Lepidopterans via COI barcoding to test for spatiotemporal global changes in PD.

Lepidopterans are a globally distributed insect clade with major economic importance as pollinators and herbivores and often high sensitivity to climate and land use changes (Habel et al. 2021; Wepprich et al. 2019). One of the three most species‐rich insect orders, they are often used as indicators of environmental quality and are found in a broad range of habitats (Goldstein 2017). Lepidopterans have also been among the most widely adopted insect orders in DNA barcode studies (Hajibabaei et al. 2006; Rougerie et al. 2014) and are consequently the second most represented insect order on the BOLD database, with over one million sequenced COI records deposited globally at the time of data accession (Ratnasingham and Hebert 2007). In this study, we use genetic data from the Barcode of Life database to calculate the PD of Lepidopteran species assemblages worldwide. From this, we explore the global patterns of Lepidopteran PD in relation to latitude, time, and environmental drivers. We replicate our analyses across six continental regions globally to further test for regional bias in spatial and temporal trends. We interpret our findings in light of both biogeographic factors and potential spatiotemporal data biases within BOLD.

## 2 Methods

### 2.1 Data Collection and Selection Criteria

Global Lepidopteran COI‐5P sequence data were downloaded via the BOLD database (Ratnasingham and Hebert 2007) and Global Biodiversity Information Facility (GBIF) (The International Barcode of Life Consortium 2016) using the rgbif package (Chamberlain et al. 2022) for R (R Core Team 2020). We then filtered the data to exclude entries according to missing species names, GPS coordinates, date of collection and sequences > 700 bp or < 600 bp, as the size of the COI barcode fragment lies within this range, so accessions with longer sequences are unlikely to represent community barcoding studies (Folmer et al. 1994), while shorter sequences representing mini‐barcodes are less likely to be reliable for phylogenetic distance estimation (Guo et al. 2022). Sequences with missing species names were removed to avoid potential problems when mapping Barcode Index Numbers to species; such issues can arise due to sample contamination and uncertain knowledge of and regional variation in intraspecific genetic distances (Cheng et al. 2023). Records were further cleaned to ensure that coordinates reflect sampling sites and not country centroids. Local assemblages were defined based on shared WGS coordinates (within 2 decimal places, or approximately 1 km) within the same year. The data were further cleaned to remove duplicate species within each local assemblage and assemblages consisting of fewer than 10 species. Each assemblage therefore represents a group of COI barcode sequences, which were obtained solely from genetic samples collected at the same place and time, although not necessarily representative of all species living in the location at the time, and which were used directly to estimate PD (see below). Resulting assemblages do not necessarily reflect functioning communities per se, given the vast diversity of Lepidopteran life histories and microhabitats, but nonetheless allow us to define local scales of potentially interacting species, to determine how such assemblages might have changed over space and time.

To test environmental drivers, records of mean annual temperature and mean annual precipitation at the location of each community location were obtained from WorldClim (Library et al. 2017) using the Raster package from R (Van Etten et al. 2022) at a spatial resolution of 2.5 arc sec. Land cover values were obtained from the Global Land Cover Dataset (Tateishi et al. 2014) using the R package ‘tmap’ (Tennekes 2018). Eighteen land cover categories were extracted for the Lepidopteran spatial points using the stars package (Pebesma 2022). Elevation of sampling sites in meters was further extracted using the elevatr package for R (Hollister 2023).

### 2.2 Alignment and Construction of Phylogenetic Tree

COI‐5P sequences from an outgroup of the order Diplopoda were downloaded from the BOLD database and were added to the sequence data to root the phylogenetic tree. The sequences were then aligned using the multiple sequence alignment program MAFFT (Katoh and Standley 2013), with an automatic alignment strategy to select the appropriate technique for the data, a gap opening penalty of 5 and a gap extension penalty of 3. The output alignment fasta file was then used to construct a maximum likelihood phylogenetic tree with FastTree (Price et al. 2010) using a general time reversible model (GTR) (Tavaré 1986) and a CAT approximation of rate heterogeneity was used for nucleotide substitution to create the maximum likelihood tree (Stamatakis 2006). FastTree's high accuracy and computational efficiency make it ideally suited to characterising broad‐scale PD metrics across large sequence datasets (Price et al. 2010). This approach is further suited to our question as we are not aiming to determine precise branching patterns but instead to estimate overall relatedness levels within communities. Given that FastTree employs a ‘minimum evolution’ principle in determining topology, any topology errors are unlikely to lead to upwardly biased PD estimates, compared to topology uncertainties or errors produced by other methods.

### 2.3 Phylogenetic Diversity Metrics

Like species richness estimators, unstandardised PD estimates may be heavily influenced by both sample size and species richness (Thompson et al. 2015; Miller et al. 2017), and the dataset used in this analysis has a large variation in both of these factors. To mitigate these potential sources of bias, we compared the mean phylogenetic distance (MPD) among all pairs of species within an assemblage using the standardised effect size of MPD, ses.mpd, calculated using the Picante package (Kembel et al. 2010). Ses.mpd compares the difference in observed mean pairwise phylogenetic distance within an observed assemblage to a randomised assemblage. To compute ses.mpd, we created an assemblage data matrix with assemblages as rows and species in the columns. Using the phylogenetic distance matrix and the assemblage data matrix, ses.mpd was calculated for each of our 4728 globally distributed assemblages, with 99 tip label randomisations used to generate null expectations for each. Positive values of ses.mpd represent phylogenetic overdispersion, whereas negative values reflect clustering. Since our dataset exhibited high variation in both sampling effort and regional species richness differences, ses.mpd is a highly appropriate metric as it has been shown to be independent of these factors (Miller et al. 2017). As an additional check on the reliability of ses.mpd, we re‐ran our analyses using another PD metric that is also robust to uneven sampling, Faith's diversity (FD) under rarefaction (rareFD). We calculated rareFD using the function phylorare (Nipperess and Matsen 2013) within the ape package for R (Paradis et al. 2004). Faith's diversity is described as the sum of the branch lengths of the phylogenetic tree (Faith 1992). Rarefaction allows for the calculation of diversity for a given number of samples based on the construction of rarefaction curves (Hurlbert 1971). We set the number of samples used for each rarefied assemblage to 10, reflecting our minimum assemblage size cut‐off. This provides an alternative approach for the comparison of FD values between assemblages with different sample sizes and underlying species richness values. Results using rarefied FD were highly congruent to those using ses.mpd, thus we report only the latter here.

### 2.4 Statistical Analysis

To investigate the global patterns of PD, linear models with a Gaussian distribution were used with the phylogenetic metrics, ses.mpd and rareFD, as the response variables and the following explanatory variables: land cover, absolute latitude, hemisphere (N or S), mean annual temperature, mean annual precipitation, elevation and sampling year. Interaction effects of environmental factors and latitude with sampling year were also investigated. Variance inflation factors were used to determine non‐collinearity of predictor variables (see also Figure S1). Model residuals exhibited significant spatial autocorrelation (Figure S2). Therefore, a random effect with a Matérn spatial smoother was run using the spaMM package for R (Rousset and Ferdy 2014). To control for temporal autocorrelation across years within sites, an additional random effect was fit for each decade/site combination. However, the vast majority of locations in the dataset were sampled only once (Figure S3). To examine whether temporal patterns are influenced by the threshold date of 2005, when the BOLD database was established (www.boldsystems.org), we further examined global temporal trends since 2005. Patterns limited to these more recent years are congruent with our main reported results (see Table S1, Figure S4). Akaike's information criterion (AIC) (Anderson et al. 1998) was used to select for the full or reduced model with the best predictive value. Marginal effects of fixed predictor variables were plotted using the ggpredict function from the ggeffects package (Lüdecke 2021) with the viridis colour palette (Garnier et al. 2024). World maps are from the package rnaturalearth (South 2017). Additional packages used included Tidyverse (Wickham et al. 2019), car (Fox et al. 2021), Lattice (Sarkar 2008), stars (Pebesma 2022) and labdsv (Roberts 2019).

Despite that standardised PD metrics are robust to uneven sampling depth across locations and times, they may still be prone to influence by other sampling biases. For instance, sampling might be more biased towards some taxonomic groups in some locations than others (e.g., use of light traps vs. malaise traps), and this could bias regional or temporal variation in standardised PD estimates (Goldstein et al. 2024). In addition, the BOLD database represents an *ad hoc* collection of barcode sequences from disparate studies with differing underlying study aims. For instance, researchers may have selected species for barcoding based on their morphological similarity or dissimilarity, and these decisions could vary among regions or change through time, biasing temporal or spatial trends. Unfortunately, we lack clearly accessible documentation in most synthesis databases (and not just within BOLD) of underlying researcher motivations and decisions. To address potential variation in such sampling biases, therefore, we therefore subset our global dataset into six continental regions (Figure 2) and conduct replicate analyses for how latitude, year and environments predict our within‐community ses.mpd estimates, again correcting for spatial and temporal autocorrelation within regions. Because sampling schemes, with their associated biases, are highly regional (Hughes et al. 2021), parallel environmental and spatial PD gradients across multiple of these six regions provide additional validation of global patterns and drivers. Differences among regions may also however reflect alternative ecological and anthropogenic processes shaping changing PD patterns differently across global biomes.

## 3 Results

**FIGURE 1:**
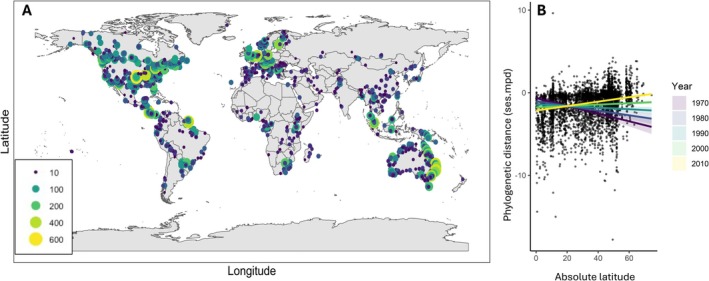
Global patterns of Lepidopteran communities. A: Spatial variation in sampling, with colours and sizes of points corresponding to the total number of taxa sampled per community. B: Global patterns of phylogenetic diversity (PD), corrected for sampling depth (see Methods text) exhibit a significant latitude x year interaction, with global Lepidopteran PD increasing through time at higher latitudes (Table S1).

Of the 956,233 globally distributed Lepidopteran COI sequences that were downloaded from GBIF and BOLD, there were 374,749 sequences remaining in the dataset after cleaning, spanning 23,156 species and 4728 assemblages (location by year combinations; Figure 1a). While many regions are well covered, there are also areas of geographical bias, notably with fewer data within parts of Africa, South America and Central Asia.

Global variation in ses.mpd was best explained by a model that included latitude, year and their interaction (Table S1). Generally, equatorial barcode‐based sampling events have remained unchanged in their represented levels of PD and tend to exhibit slight phylogenetic underdispersion overall. High latitude assemblages, in contrast, have become much more diverse in recent decades, flipping from strongly underdispersed to moderately overdispersed through time (Figure 1b). None of the originally included environmental variables improved model fit or were included in the final global model, either alone or in interaction with year.

**FIGURE 2:**
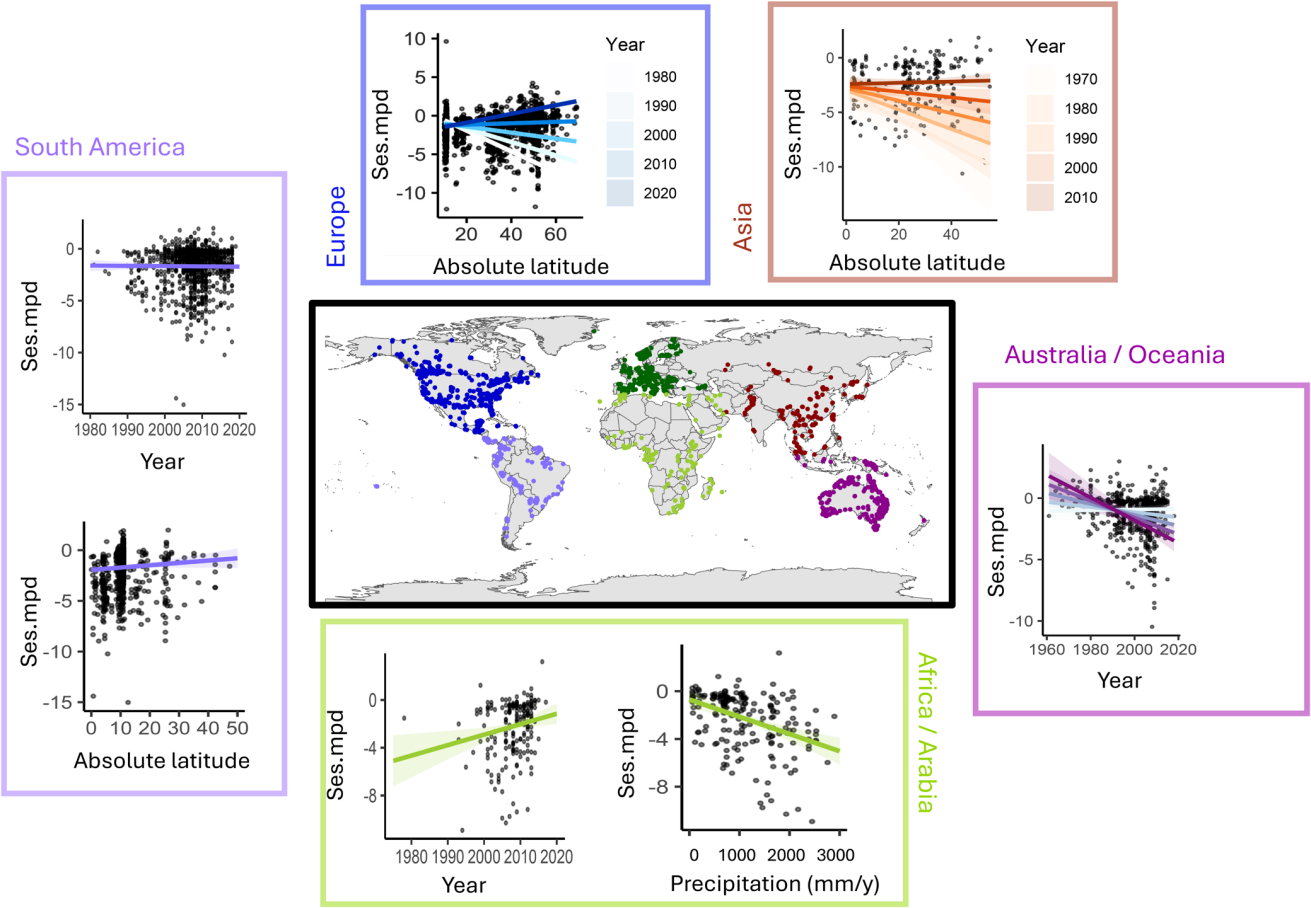
Regional drivers of phylogenetic diversity (PD). Plots depict partial fixed effects remaining in best fit models of spatiotemporal variation in PD separately by geographical region (with shading representing standard errors). The models from which these estimates are derived are found in Table S1.

The global pattern of PD in space and time shows parallelism across different regional gradients (Table S1), with patterns in both North America and Asia closely matching (and likely influencing) the global latitudinal trends. Also conforming to the general pattern, both South America and Europe exhibit a positive relationship of PD with (absolute) latitude, and Europe exhibits a positive influence of time, particularly in lowland areas. Similarly in Africa we find evidence of PD increasing through time. While not evident at the global scale, two regions show parallel cases of decreasing PD in higher‐rainfall areas: both in Africa/Arabia at all time points, and in Australasia wetter regions show significant declines in PD towards the present. In South America, the best model includes a weak and non‐significant overall decline in PD towards the present. Together these results paint a picture of expanding PD in high latitude, Northern Hemisphere regions and temporal declines in PD in more tropical and equatorial areas (Table S1, Figure 2).

After accounting for spatial autocorrelation, land use was eliminated as an explanatory variable from all models, although we did not test targeted hypotheses about particular land use categories. The accuracy of our GPC coordinates (2 decimals) may not be suitable for detecting fine‐scale landscape impacts acting at the scale of meters.

## 4 Discussion

We evaluated changes in global Lepidopteran PD in relation to time, spatial distribution, land cover and climate. We find that standardised estimates of Lepidopteran PD have increased through time at high latitudes, with contemporary processes thus reversing the historical pattern of latitudinally declining PD observed at the earlier time points in our data (Figure 1b). All of our assemblages result from barcoding sampling, and changes in how insects are captured and processed may have contributed to this pattern (however, we do not see significant changes in PD through time in equatorial regions). To investigate the extent to which regional sampling might have influenced findings, and to tease apart regional nuances, we repeated our analyses across six global regions and find parallel results that are therefore robust to regional sampling variation. Because it is likely that DNA barcoding sampling strategies have varied through time and across regions, potentially influencing some of our findings, we therefore express more confidence in the results that exhibit highest parallelism across regions.

Previous studies have reported both latitudinally increasing or decreasing PD across spatial gradients (see further discussion below), although many of these do not control for species richness when estimating PD, and therefore confound different facets of biodiversity (as uncorrected PD will be upwardly biased in more species‐rich areas). Moreover, while one previous study shows strong fluctuations in PD at high latitudes over geological time scales, mirroring our findings (Bhatta et al. 2024), ours is the first study of which we are aware to document dynamic reversals in macroecological biodiversity gradients on contemporary time scales.

The recent increasing PD at high latitudes also corresponds to recent findings of (sometimes dramatic) increases in species richness within high latitude Lepidopteran communities (Franzén et al. 2023), despite Lepidopteran species richness typically following the traditional biodiversity gradient, exhibiting decreasing species richness with increasing latitude (Hawkins 2010). Our observed pattern of a recent reversal in the global latitudinal cline in Lepidopteran phylogenetic dispersion could result from the biogeographic history of lineages (Davies and Buckley 2011) coupled with patterns of phylogenetic inheritance of colonisation‐related traits. Specifically, Lepidoptera are thought to have originated in the tropics where they currently have the highest number of extant species (Levin 2001) and have dispersed more recently towards more temperate regions and higher latitudes (Jablonski et al. 2006; Hawkins and DeVries 2009). However, global patterns of PD, and especially recent dynamics in observed clines, are also impacted by how colonists are filtered by latitude during historical and contemporary range shifts. If species dispersing to higher latitudes during post‐glacial or contemporary range expansions do so on the basis of phylogenetically conserved traits (such as cold tolerance), then higher latitude communities may be more closely related than expected, resulting in a pattern of decreasing PD with latitude, in parallel to species richness patterns (as has been observed in herbaceous plants, ants and vertebrates (Economo et al. 2018; Gaüzère et al. 2022; Massante et al. 2019), but in contrast to our own findings). Alternatively, if the traits predicting successful range expansion are not well conserved phylogenetically, ecological filtering along the colonisation gradient could result in higher than expected PD in higher latitude assemblages, reflecting the phylogenetically disparate nature of colonising species.

In Lepidopterans, where we here observe increased phylogenetic overdispersion with latitude in recent decades, recent climate‐mediated range shifts have been shown to correlate with ecological generalism (Platts et al. 2019; Pöyry et al. 2009), which is notoriously highly evolutionarily labile in this group (Bernays and Graham 1988; Nylin et al. 2014; Oppenheim et al. 2018). Range‐expanding Lepidopterans have further been shown to develop broader diet breadth and decreased habitat specificity at the leading edge of their range shifts (Oliver et al. 2009; Lancaster 2020). If the evolution of these generalist characteristics and associated range expansions arises independently, multiple times in the Lepidopteran lineage, these phylogenetically independent, eco‐evolutionary dynamics may explain our observed macroecological pattern of recent phylogenetic overdispersion in high latitude assemblages (Lancaster 2022). This putative process of dynamic range shifts contributing to rapid temporal increases in PD at high latitudes is also supported by traditional concepts of tropical niche conservatism and its role in determining the distribution of phylogenetic diversity gradients, whereby clades originating and remaining in the tropics exhibit a greater phylogenetic inertia compared to clades that have members dispersing to higher latitudes (Wiens and Donoghue 2004; Hawkins and DeVries 2009; Hawkins et al. 2014; Chazot et al. 2021).

Our interpretation of parallel, temporal increases in PD at high latitudes implicates widespread range shifts to cooler climates in the Anthropocene (Lenoir et al. 2020) as a causal driver of global variation in Lepidopteran PD and its changing patterns. This interpretation requires additional work investigating the taxa contributing most strongly to PD change overall. Our interpretation is supported by our observation that the strongest interactions of time with latitude in explaining spatiotemporal patterns of PD are observed in previously glaciated regions in the Northern Hemisphere (Figure 2), regions which also observe greater rates and magnitudes of historical and recent range expansions (Hewitt 1999, 2000).

In terms of climate, temperature surprisingly appeared to play no role in determining either global or regional patterns of PD, after correcting for spatial autocorrelation of environments. That broad‐scale pattern of climate coincide strongly with latitude and elevation means that it is often difficult to disentangle spatial and climatic drivers of biotic clines. Elevational patterns were also generally lacking, except in Europe, where a historic elevational cline, of higher PD at higher elevations, has recently been dampened. A similar pattern of increasing PD with elevation was also found in geometrid moths in China, with phylogenetic diversity increasing with increasing altitude, while species richness was decreasing. This pattern was attributed to preferential colonisation of higher elevations by habitat generalists, which were phylogenetically more distinct from each other than were members of the more species‐rich, lower elevation assemblages from which they were derived (Zou et al. 2016), a result which strongly mirrors ours, despite the use of very differing data sources. In our data, it is unclear why the elevational patterns found in Europe were not generally apparent in other countries, or why the European elevational cline appears to have dampened dramatically in recent years. One explanation, supported by our findings on latitudinal trends, may be that latitudinal range shifts and processes associated with recent biotic homogenisation of lowland habitats have enabled lowlands to ‘catch up’ based on the recent influx of diverse, warm‐adapted generalists (Burton 2003; Álvarez et al. 2024).

Finally, it is alarming to observe in our data recent declines in PD through time in tropical (higher‐rainfall) regions of the Global South (Figure 2). Previous studies conducted on a local scale have found that human impacts in tropical forests can decrease arthropod PD, in contrast to levels observed in primary forest sites (Hoenle et al. 2024). Arthropods in general may show lower resistance to recovery from deforestation than other groups such as birds, whose patterns of PD can be less impacted by disturbance (Edwards et al. 2017). Of course we also cannot rule out the possibility that at a global scale, tropical (but not temperate) ecologists are increasingly choosing to barcode more closely related species from within their sampling events than they did in former years. More accessible metadata on sampling decisions within global synthesis databases such as BOLD would help resolve this problem for future biodiversity modellers.

Our results indicate the need to consider other dimensions of biodiversity in addition to species richness when inferring patterns of biodiversity at a global scale. In a changing and developing world, effective conservation strategies should consider the species, functional and evolutionary processes in an integrative approach to biodiversity assessment (Devictor et al. 2010; Cai et al. 2018). An understanding of how PD changes over latitudinal gradients and range shifts can be useful in predicting future local biodiversity and species community structure under climate change (Fitt and Lancaster 2017), especially where patterns of PD predict ecological dynamics in novel assemblages shaped by range shifting and changes in local habitat specificity. Monitoring of global change in PD can further be used to enhance conservation strategies to preserve evolutionary history and genetic distinctiveness globally (Isaac et al. 2007). Amidst the increased accessibility to molecular techniques, such as DNA barcoding, more spatially comprehensive COI barcode data will continue to become available for more taxonomic groups, providing opportunity for further analysis. Since we downloaded the BOLD records for our analysis in 2022, the number of sequence accessions available for Lepidoptera has more than doubled, exhibiting a hyper‐acceleration of genetic biodiversity assessment. Combined with recent improvements in data deposition pipelines, quality control and metadata within BOLD (Ratnasingham et al. 2024), this data deluge brings challenges but also perhaps one of our best resources for future reanalysis to continue tracking these global trends.

## Author Contributions

**Jillian Muirberry:** conceptualization (equal), data curation (lead), formal analysis (lead), investigation (lead), visualization (equal), writing – original draft (lead), writing – review and editing (supporting). **Lesley T. Lancaster:** conceptualization (equal), formal analysis (supporting), investigation (supporting), project administration (lead), supervision (lead), visualization (equal), writing – original draft (supporting), writing – review and editing (lead).

## Funding

This work was supported by the Natural Environment Research Council (NE/N008499/1).

## Ethics Statement

This work does not require ethical approval, involve patents or clinical trials or reproduced materials.

## Conflicts of Interest

The authors declare no conflicts of interest.

## Supporting information


**Data S1:** ece372557‐sup‐0001‐Supinfo1.csv.

**Figure S1:** Correlation plots of the phylogenetic diversity metric (ses.mpd) and continuous predictors. Diagonal shows histograms of their distributions. Upper panels show pairwise scatterplots with a lowess line fitted.
**Figure S2:** Correlogram of model residuals (see global model, table S1), before (A) vs. after (B) correcting for spatial autocorrelation of the data.
**Figure S3:** Most communities were sampled only once. (A) Per site and (B) per decade/site combination, the histogram X‐axis indicates the total number of sampling events, while the Y‐axis shows the number of sites (A) or decade/site combinations (B) exhibiting that sampling depth.
**Figure S4:** Latitudinal and temporal patterns in the standard effect size of mean pairwise phylogenetic distances within sampled assemblages, with data truncated to remove records dating to pre‐2005.
**Table S1:** Statistical results: fixed effects in best fit models, explaining variation in Ses.mpd (standardised Phylogenetic Diversity) at global and regional scales.

## Data Availability

This work does not rely on original data. All derived data are uploaded as article supporting material.
