# Supplementary material for: Global Changes in Lepidopteran Phylogenetic Diversity Across Space and Time
Source: Ecol Evol. 2026 Jan 12;16(1):e72557. doi: 10.1002/ece3.72557 (PMC12795615; doi:10.1002/ece3.72557)
Supplement: Supplementary file 2 — Figure S1: Correlation plots of the phylogenetic diversity metric (ses.mpd) and continuous predictors. Diagonal shows histograms of their distributions. Upper panels show pairwise scatterplots with a lowess line fitted. Figure S2: Correlogram of model residuals (see global model, table S1), before (A) vs. after (B) correcting for spatial autocorrelation of the data. Figure S3: Most communities were sampled only once. (A) Per site and (B) per decade/site combination, the histogram X‐axis indicates the total number of sampling events, while the Y‐axis shows the number of sites (A) or decade/site combinations (B) exhibiting that sampling depth. Figure S4: Latitudinal and temporal patterns in the standard effect size of mean pairwise phylogenetic distances within sampled assemblages, with data truncated to remove records dating to pre‐2005. Table S1: Statistical results: fixed effects in best fit models, explaining variation in Ses.mpd (standardised Phylogenetic Diversity) at global and regional scales. [file ECE3-16-e72557-s001.docx]

Supplementary information *Global changes in Lepidopteran phylogenetic diversity across space and time*

Figure S1: Correlation plots of the phylogenetic diversity metric (ses.mpd) and continuous predictors. Diagonal shows histograms of their distributions. Upper panels show pairwise scatterplots with a lowess line fitted.

Figure S2: Correlogram of model residuals (see global model, table S1), before (A) vs. after (B) correcting for spatial autocorrelation of the data.

Figure S3: Most communities were sampled only once. (A) Per site, and (B) per decade/site combination, the histogram X-axis indicates the total number of sampling events, while the Y-axis shows the number of sites (A) or decade/site combinations (B) exhibiting that sampling depth.

Figure S4: Latitudinal and temporal patterns in the standard effect size of mean pairwise phylogenetic distances within sampled assemblages, with data truncated to remove records dating to pre-2005.

Table S1: Statistical results: fixed effects in best fit models, explaining variation in Ses.mpd (standardised Phylogenetic Diversity) at global and regional scales.

| Model | Variable | Estimate | lower 95% CI | upper 95% CI | Cond. SE | F | P |
| --- | --- | --- | --- | --- | --- | --- | --- |
|  |  |  |  |  |  |  |  |
| *Global (all years)* | Intercept | 85.82 |  |  | 17.48 |  |  |
|  | Absolute Latitude | -3.71 | -4.76 | -2.66 | 0.54 | 50.75 | <00001 |
|  | Year | -0.04 | -0.061 | -0.027 | 0.0087 | 28.9 | <00001 |
|  | Abslat * Year | 0.002 | 0.001 | 0.002 | 0.00027 | 51.94 | <00001 |
|  |  |  |  |  |  |  |  |
| *Global (since 2005)* | Intercept | 81.55 |  |  | 2.41 |  |  |
|  | Absolute Latitude | -7.28 | -9.38 | -5.18 | 1.07 | 46.25 | <00001 |
|  | Year | -0.04 | -0.075 | -0.0091 | -2.50 | 6.26 | 0.01 |
|  | Abslat * Year | 0.004 | 0.003 | 0.005 | 0.00005 | 46.78 | <00001 |
|  |  |  |  |  |  |  |  |
| *Regional – all years* |  |  |  |  |  |  |  |
|  |  |  |  |  |  |  |  |
| Asia | Intercept | 5.68 |  |  | 66.41 |  |  |
|  | Absolute Latitude | -6.17 | -11.88 | -0.46 | 2.91 | 4.48 | 0.035 |
|  | Year | -0.004 | -0.069 | 0.061 | 0.033 | 0.014 | 0.9 |
|  | Abslat * Year | 0.003 | 0.0002 | 0.006 | 0.0014 | 4.46 | 0.036 |
|  |  |  |  |  |  |  |  |
| Austral- | Intercept | -308.4 |  |  | 96.77 |  |  |
| asia | Precipitation | 0.097 | 0.018 | 0.18 | 0.04 | 5.78 | 0.017 |
|  | Year | 0.152 | 0.058 | 0.25 | 0.048 | 9.96 | 0.002 |
|  | Precipitation * Year | -0.000048 | -0.00009 | -0.000009 | 0.00002 | 5.74 | 0.018 |
|  |  |  |  |  |  |  |  |
|  | Intercept | -127.5 |  |  | 28.6 |  |  |
|  | Absolute Latitude | 0.0923 | 0.064 | 0.12 | 0.0146 | 40.21 | <0.0001 |
|  | Elevation | 0.0639 | -0.006 | 0.13 | 0.0357 | 3.49 | 0.06 |
|  | Year | 0.061 | 0.033 | 0.09 | 0.0139 | 18.65 | <0.0001 |
|  | Elevation * Year | -0.0000318 | -7.00E-05 | 3.00E-06 | 0.0000178 | 3.48 | 0.06 |
|  |  |  |  |  |  |  |  |
| Africa/ | Intercept | -148.9 |  |  |  |  |  |
| Arabia | Absolute Latitude | 0.0293 | -0.008 | 0.066 | 0.019 | 2.38 | 0.13 |
|  | Elevation | -0.00042 | -0.001 | 0.0001 | 0.000272 | 2.38 | 0.13 |
|  | Precipitation | -0.00121 | -0.002 | -0.0005 | 0.000358 | 11.47 | 0.001 |
|  | Year | 0.0735 | 0.008 | 0.14 | 0.0343 | 4.83 | 0.03 |
|  |  |  |  |  |  |  |  |
| North | Intercept | 176.93 |  |  |  |  |  |
| America | Absolute Latitude | -10.15 | -12.05 | -8.25 | 0.971 | 109.33 | <0.0001 |
|  | Year | -0.09 | -0.11 | -0.065 | 0.013 | 49.34 | <0.0001 |
|  | Abslat * Year | 0.0051 | 0.004 | 0.006 | 0.00048 | 110.14 | <0.0001 |
|  |  |  |  |  |  |  |  |
| South | Intercept | 42.43 |  |  |  |  |  |
| America | Absolute Latitude | 0.049 | 0.012 | 0.086 | 0.0019 | 6.89 | 0.009 |
|  | Year | -0.023 | -0.046 | -0.0003 | 0.012 | 3.95 | 0.047 |
